# Drug Repurposing Flubendazole to Suppress Tumorigenicity via PCSK9-dependent Inhibition and Potentiate Lenvatinib Therapy for Hepatocellular Carcinoma

**DOI:** 10.7150/ijbs.81415

**Published:** 2023-04-23

**Authors:** Wenjiao Jin, Junming Yu, Yang Su, Hechun Lin, Tengfei Liu, Jing Chen, Chao Ge, Fangyu Zhao, Qin Geng, Lin Mao, Shuqing Jiang, Ying Cui, Taoyang Chen, Guoping Jiang, Jinjun Li, Chunxiao Miao, Xiuying Xiao, Hong Li

**Affiliations:** 1 State Key Laboratory of Oncogenes and Related Genes, Shanghai Cancer Institute, Renji Hospital, Shanghai Jiaotong University School of Medicine, Shanghai 200032, China.; 2 Department of Oncology, Renji Hospital, Shanghai Jiaotong University School of Medicine, Shanghai 200127, China.; 3 Key Laboratory of Carcinogenesis and Translational Research (Ministry of Education), Hepatopancreatobiliary Surgery Department I, Peking University Cancer Hospital & Institute, Beijing 100142, China; 4 Cancer Institute of Guangxi, Nanning 530015, China.; 5 Qidong Liver Cancer Institute, Qidong 226299, China.; 6 Department of General Surgery, The First Affiliated Hospital, School of Medicine, Zhejiang University, Hangzhou 310003, China.

**Keywords:** Drug repurposing, Flubendazole, Hepatocellular carcinoma, PCSK9

## Abstract

Hepatocellular carcinoma (HCC) is one of the most lethal malignant cancers across the world. It has a poor prognosis and lacks effective therapies, especially for patients with advanced-stage cancer, indicating an urgent need for new therapies and novel therapeutic targets. Here, by screening the U.S. Food and Drug Administration drug library against HCC cell lines, we identified that flubendazole, a traditional anthelmintic drug, could prominently suppress HCC cells *in vivo* and *in vitro*. RNA sequence analysis and cellular thermal shift assays showed that flubendazole reduced the expression of PCSK9 protein by direct targeting. The increased expression of PCSK9 in HCC tissues was demonstrated to be correlated with poor prognosis, and the inhibitory ability of flubendazole was selectively dependent on PCSK9 expression. PCSK9 knockdown abolished the antitumor effects of flubendazole in HCC. Mechanistically, flubendazole inhibited the Hedgehog signaling pathway induced by PCSK9, resulting in the downregulation of smoothened (SMO) and GLI Family Zinc Finger 1 (Gli1). Moreover, combining flubendazole with lenvatinib was found more effective than administering lenvatinib only for HCC treatment *in vivo* and *in vitro*. These findings reveal the therapeutic potential of flubendazole against HCC and provide clues on new repurposed drugs and targets for cancer treatment.

## Introduction

Hepatocellular carcinoma (HCC) is one of the most common primary malignancies worldwide and is characterized by rapid progression and poor prognosis 1. With an increase in mortality, HCC has become the third leading cause of mortality worldwide. Surgical resection is the first choice of treatment for early stage HCC 2. However, owing to the asymptomatic early stage and lack of specific methods for early diagnosis, HCC is usually diagnosed at an advanced stage, and patients lose the opportunity for surgical resection 3. Chemotherapy and radiotherapy have shown limited efficacy in the treatment of advanced HCC. The first-line recommended drugs sorafenib and lenvatinib improves overall survival by approximately 3 months only 4,5 owing to chemotherapy resistance exhibited by the cancer cells. Therefore, new therapeutic agents or combination therapies are urgently required for effective treatment of HCC.

Drug repurposing which means repositioning of approved drugs is a new approach for the development of innovative modes of treatments with low cost and high efficiency 6,7. Recently, several existing drugs were approved for cancer prevention. Drug repurposing has provided several interesting candidate drugs that target mutations or malfunctioning receptors and proteins for HCC treatment. The anthelmintic niclosamide has been reported to have antitumor activity in HCC cell lines through a bioinformatics search 8. Chen *et al*. described the use of the antifungal agent ketoconazole, which induces apoptosis by overactivating mitophagy in HCC 9. Simvastatin, an HMG-CoA reductase inhibitor, induces G0/G1 cell cycle arrest by inhibiting the STAT3/Skp2 axis and activating the AMPK pathway 10. Screening approved drugs based on tumors or molecular targets can greatly shorten the time required for compound discovery. Simultaneously, owing to the clear pharmacokinetic properties and safety parameters of marketed drugs, they effectively reduce the risk of clinical research failure.

Flubendazole is a benzimidazole compound approved for the treatment of gastrointestinal nematode infections 11 and was first discovered as an anthelmintic in humans. In 2010, flubendazole was found to exert an inhibitory effect on leukemia and myeloma cells by suppressing microtubes 12. Subsequently, the antitumor potentials of flubendazole have been reported, including the promotion of ferroptosis in prostate cancer 13, induction of autophagy in triple negative breast cancer (TNBC) and lung cancer 14, inhibiting STAT3 15, and activation of mitotic catastrophe in colorectal cancer 16. In addition, flubendazole induces apoptosis and G2/M cell cycle arrest in glioblastoma cell lines 17,18. However, the potential effects and mechanisms of action of flubendazole in HCC remain unclear.

Here, we identified the antitumor effects of flubendazole in HCC *in vivo* and *in vitro* using drug repurposing screening for the first time. Flubendazole downregulates the expression of PCSK9, an important cholesterol metabolism factor, by directly interacting with it. PCSK9 facilitates HCC carcinogenesis and metastasis *in vivo* and *in vitro* through activation of the Hedgehog (Hh) signaling pathway via upregulation of the smoothened (SMO) and GLI Family Zinc Finger 1 (Gli1) proteins. Further, the combination of flubendazole and lenvatinib showed synergistic effects against HCC cells* in vivo* and *in vitro*. Taken together, these results demonstrate the antitumor effect of flubendazole in HCC treatment by downregulating the PCSK9-induced Hh signaling pathway and indicate new therapeutic strategies and preclinical biomarkers for HCC diagnosis and treatment.

## Methods

### Cell lines and cell culture

The human HCC cell lines SNU449, PLC/PRF/5, Hep3B, HepG2 and the human renal epithelial cell line HEK-293T were purchased from the American Type Culture Collection (ATCC, USA). Huh7 cells were purchased from the Riken Cell Bank (Tokyo, Japan). The Li7 cells were obtained from the Chinese Academy of Sciences (Shanghai, China). HCC-LY10 was retrieved and established in our lab from a Chinese patient diagnosed with primary hepatocellular carcinoma. The patient was informed and agreed with the content. MHCC-97H and MHCC-LM3 cell lines were kindly provided by Zhongshan Hospital, Fudan University. All the cell lines were detected and approved by the Cell STR (Short Tandem Repeat) identification.

### Compounds and antibodies

An FDA-approved Drug Library (L1300) was purchased from Selleck Chemicals Texas (Texas, USA). Flubendazole (B0294) and lenvatinib (10981) were purchased from MedChem Express (New Jersey, USA). Antibodies against PCSK9 (85813) and GLI1 (3538) used for western blotting were obtained from Cell Signaling Technology (Massachusetts, USA). The monoclonal antibody against SMO (66851-1-Ig) was purchased from Proteintech (Illinois, USA). The monoclonal antibody against PCSK9 (MA5-32843) was obtained from Invitrogen (California, USA).

### Drug repurposing screen

Huh7, MHCC-97H, and SNU449 cells were incubated in 96-well plates at a density of 1500-3000 cells/well. On the next day, drugs from the FDA-approved drug library were diluted to 10 μM and added to each well. Cell viability was measured using the cell counting kit-8 (CCK-8) assay at 24, 48, and 72 h after treatment. All drugs were screened in duplicate.

### Enzyme linked immunosorbent assay (ELISA)

Enzyme-linked immunosorbent assay was performed using the Human Proprotein Convertase 9/PCSK9 DuoSet ELISA kit, according to the manufacturer's instructions (R&D Systems, Minnesota, USA). Briefly, a 96-well microplate was coated with diluted capture antibodies on the first day. The next day, the plate was blocked with reagent diluent, and the serum samples (1:10 dilution) or cell supernatant was added to the well and incubated for 2 h. Next, detection antibodies and streptavidin-horseradish peroxidase were added to the plates and incubated at room temperature for 20 min. The results were analyzed using a spectrophotometer at a wavelength of 450 nm. Serum samples from patients with HCC were provided by the Qidong Liver Cancer Institute (Qidong, China), while samples from healthy individuals were obtained from the physical examination collections of the Shanghai Cancer Institute. The patient was informed of and agreed to participate in the study.

### Flow cytometry

The percentage of apoptotic cells was determined by flow cytometry using a PE Annexin V Apoptosis Detection Kit I (BD 559763, New Jersey, USA) according to the manufacturer's instructions. The cell cycle stage of cell lines was detected by flow cytometry as described previously 19. Briefly, the cells were incubated and treated with flubendazole or DMSO for 72 h. After harvesting with trypsin, the cells were washed by PBS (phosphate buffered solution) and fixed with 70% ethanol for 12 h at 4 ℃. The fixed cells were then washed with PBS and stained with propidium iodide (Sigma-Aldrich, USA) at 4 ℃ for 30 min. Cells were dissociated into single-cell suspensions, and cell cycle distribution was measured and analyzed by flow cytometry.

### Plasmid vectors and lentivirus packaging

Short hairpin RNAs (shRNAs) targeting PCSK9 (shPCSK9#1 and shPCSK9#2) was purchased from Horizon Discovery (Cambridge, United Kingdom) along with the negative control vector pLKO.1. The GV365-PCSK9 and negative control vectors were obtained from GeneChem (Shanghai, China). Lentivirus packaging and plasmid transfection were performed according to the manufacturer's instructions (PolyPlus). The sequences are listed in Table S1.

### Xenograft mouse models

All animal care procedures and experiments were approved by the Shanghai Cancer Institute Animal Care Committee and Shanghai Medical Experimental Animal Care Commission. To determine the antitumor effect of flubendazole, 2.5×10^6^ Huh7 and 3×10^6^ MHCC-97H cells were injected subcutaneously into the right posterior flank of 6-8-week-old female BALB/c nude mice (*n* = 6 or 8 per group). After the tumor volume reached approximately 100 mm^3^, the mice were randomly divided into two groups and treated with control or flubendazole (40 mg/kg) administered via peritoneal injection. The tumor size and weight of the mice were measured thrice per week. The tumor volume was calculated using the following formula: tumor volume equals ½ length × width^2^. When the tumor size increased to approximately 2000 mm^3^, the mice were sacrificed using CO_2_. For the orthotopic liver transplantation model, we engrafted 1×10^6^ HCC cells transfected with shPCSK9#1or the NC vector into the left hepatic lobe of the 4-5-week-old male BALB/c nude mice (*n* = 6 or 8 per group). After 5-8 weeks, the mice were sacrificed, and the tumor tissue and liver were harvested to determine the metastatic nodules by hematoxylin and eosin (H&E) staining. All animals were maintained under specific-pathogen free (SPF) conditions.

### RNA extraction and Quantitative Real-Time PCR

Total RNA was isolated from treated HCC cells using TRIzol reagent (Invitrogen, California, USA). The cDNA was reverse-transcribed using the PrimeScript RT Reagent Kit (Takara, Japan) according to the manufacturer's instructions. Relative mRNA levels were determined using TB Green Premix Ex Taq II (Takara, Japan), according to the manufacturer's instructions. The primer sequences are listed in Table S2.

### Western blotting

Proteins from treated cells were extracted using RIPA lysis buffer (Thermo Fisher Scientific, Massachusetts, USA) with protease and phosphatase inhibitor cocktails (Roche, Basel, Switzerland). Protein samples were subjected to SDS-PAGE and transferred onto polyvinylidene fluoride (PVDF) membranes. The membranes were blocked with SuperBlock Blocking Buffer (Thermo Fisher Scientific) for 1 h at 25 °C and incubated with primary antibodies overnight. The next day, the membranes were incubated with secondary antibodies for 2 h. Specific protein bands were detected by chemiluminescence methods using Pierce™ ECL Western Blotting Substrate (Thermo Fisher Scientific, Massachusetts, USA). The concentrations and dilutions of antibodies used for western blotting are listed in Table S3.

### Cellular thermal shift assay (CETSA)

A cellular thermal shift assay was performed according to the manufacturer's instructions 20. For the HCC-LY10 cell lysates, the cells were collected and washed twice with cold PBS. The cell pellets were subsequently resuspended in RIPA lysis buffer (Thermo Fisher Scientific, Massachusetts, USA) containing protease inhibitor cocktails (Roche, Basel, Switzerland). The cell lysates were centrifuged at 20,000 *g* for 15 min at 4 °C and the supernatants were collected. The soluble protein supernatants were incubated with DMSO or 16 μM flubendazole for 1 h at room temperature. After heating to 55 - 73 °C, the samples were incubated at room temperature for 3 min. For intact HCC cells, the cells were directly treated with DMSO or 16 μM flubendazole for 2 h at 37 °C and 5% CO2. The cells were then collected and heated to 55 - 73 °C for 5 min and at room temperature for 3 min. Thereafter, the cells were lysed using the freeze-thaw method. The heated lysates or intact cells were centrifuged at 20,000 *g* for 20 min at 4 °C and the supernatants were collected for western blotting.

### Molecular docking

The crystal structure of PCSK9 was obtained from the RCSB Protein Data Bank (RCSB PDB). The 3D structure of flubendazole was obtained from PubChem Data Bank. Molecular docking was performed using AutoDock Vina software to calculate the potential interactions between PCSK9 and flubendazole 21,22. PCSK9 and flubendazole were prepared using AutoDock Tools. The final graphic results were analyzed using Pymol software.

### Fluorescence microscopy

NBD-cholesterol (Thermo Fisher Scientific, Massachusetts, USA) was solubilized in DMSO and diluted to a final concentration of 16 µmol/L with fresh medium. HCC cells were seeded in 6-well plates and treated with flubendazole for 72 h. Thereafter, the medium was removed and incubated with NBD-Cholesterol solution for 1 h at 37 °C and 5% CO_2_ in the dark. After incubation, the cells were washed with PBS and observed under an Olympus microscope (CKX53) using an FITC filter.

### Immunofluorescence

Cells were incubated with NBD-cholesterol as described above in a Lab-Tek Chamber Slide Cover (Thermo Fisher Scientific, Massachusetts, USA). The cells were fixed with 4% formaldehyde (Sigma-Aldrich, USA) for 20 min in the dark at 25 °C and infiltrated with 0.5% Triton X-100 for 10 min. The cells were subsequently blocked and incubated with SMO antibody (1:100 dilution, Proteintech, Illinois, USA) overnight at 4 °C. The next day, the wells were first incubated with Alexa Fluor® 594-conjugated goat anti-mouse secondary antibodies for 1 h and later with Hoechst 33342 (Beyotime Biotechnology, China) for 30 min. The coverslips were observed under a confocal laser scanning microscope (Leica TCS-SP5, Leica Microsystems, Wetzlar, Germany).

### Immunohistochemistry

HCC tissues were obtained from patients receiving surgical treatment at the Qidong Liver Cancer Institute (Qidong, China). All patients provided written informed consent. Tissue microarrays containing 236 HCC tissue samples were analyzed using immunohistochemistry (IHC) as described previously 23. In brief, the samples were incubated with PCSK9 (1:250) and SMO (1:500) antibodies overnight at 4 °C. The next day, the microarrays were incubated with secondary antibodies and stained. Images were captured using the Axioskop 2 microscope (Carl Zeiss, Oberkochen, Germany). IHC staining was scored as 0-4 according to the staining intensity and the percentage of positive cells, as described previously 23, and was confirmed blindly by two pathologists. Based on these scores, patients with HCC were divided into two groups: low expression (scores 0-2) and high expression (scores 3-4). Detailed information on the antibodies used for Immunohistochemistry is listed in Table S3.

### Clonogenic assays

HCC cells (1500-3000) were seeded in 6- or 12-well plates. Drugs were added to the plates after 24 h of incubation and cultured for 7-14 d. When the cells grew into colonies of suitable size, they were fixed with 10% formalin and stained with crystal violet (Sango Biotech, China). The number of colonies was calculated using the ImageJ software.

### Transwell migration and invasion assay

HCC cells (1×10^5^) were added to Transwell chambers with culture medium (8 µm, Corning, New York, USA). The bottom layer of the membrane was suspended in a medium supplemented with FBS. After 12-24 h, cells were fixed and stained with crystal violet. The number of transmembraned cells was determined using ImageJ software.

### RNA sequencing and data analysis

Huh7, MHCC-97H, and SNU449 cells treated with 0.5 μM flubendazole or DMSO for 72 h were harvested using TRIzol and subjected to mRNA Sequence Analysis (Sinotech Genomics, Shanghai). Total RNA was purified using the Tianmo#TR205-200 kit. The quality of total RNA was determined by Agilent Bioanalyzer 2100 (Agilent technologies, California, USA) and quantified by Qubit®3.0 Fluorometer and NanoDrop One Spectrophotometer. Enrichment of differential gene analysis for KEGG and gene ontology (GO) pathways was performed using GSEA software.

### Quantification Cholesterol Assay

Cholesterol extraction was performed as described before 24. The quantification of cholesterol in HCC cells were conducted using Amplex® Red Cholesterol Assay Kit (Invitrogen, California, USA) according to the manufacturer's instructions.

### Statistics

All the results are presented as mean ± SEM and all the *in vitro* experiments were conducted at least for three replicates. Data from two groups were compared using an unpaired two-tailed Student's* t* test. Comparison of data for more than two groups was conducted using analysis of variance. Survival curves were obtained using the Kaplan-Meier method. Correlation analysis between the two genes was performed using the Spearman correlation analysis. The IC50 value of flubendazole was calculated using the logit method. Coefficient of drug in interaction (CDI) was calculated using the following equation:

CDI = AB / A × B

AB represents the viability of cells treated with a combination of the two drugs, and A and B represent the viability of cells treated with a single drug. A CDI < 1 indicates that the combination of the two drugs has a synergistic effect, whereas a CDI = 1 indicates an additive effect. All data were analyzed using Graphpad prism 7.5. * represents *P* < 0.05 which means the differences were statistically significant, ** represents* P* < 0.01, *** represents* P* < 0.001.

## Results

### Flubendazole blocks tumor proliferation of HCC* in vitro* and *in vivo*

**Figure 1:**
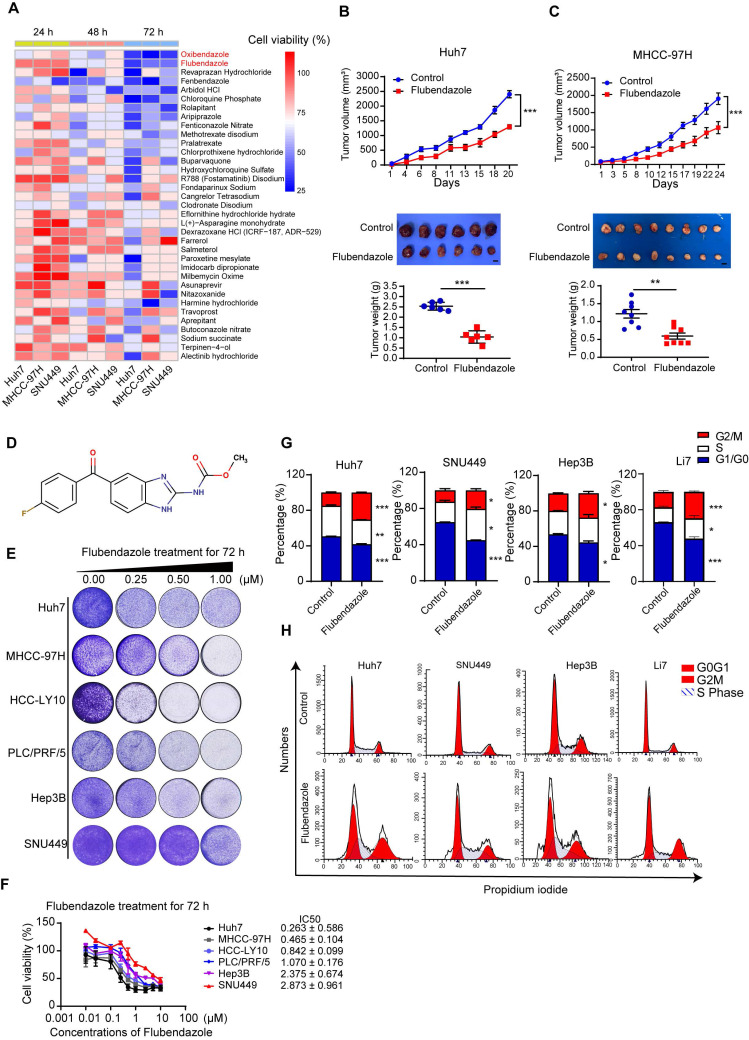
** Flubendazole blocks tumor proliferation of HCC *in vitro* and* in vivo*.** (A) Cell viabilities of Huh7, MHCC-97H and SNU449 cells treated with 10 µM FDA approved drugs for 24, 48, 72 hours. (B), (C) The upper parts show the subcutaneous tumor volumes of Huh7 (*n* = 6) and MHCC-97H (*n* = 8) in nude mice treated with control (DMSO) or flubendazole (40 mg/kg) by peritoneal injection. The lower parts show the tumor images and weights of nude mice treated with control (DMSO) or flubendazole. Scale bars, 1 cm. (D) Chemical structure of flubendazole. (E) Colony formation assays of HCC cells treated with 0.25, 0.50, 1.00 µM flubendazole for 72 hours. (F) Cell viabilities and IC50 of HCC cells treated with different concentrations of flubendazole for 72 hours. (G) and (H) Representative images and quantification of HCC cells treated with control, 0.25 or 0.5 µM flubendazole for 72 hours. Cells were then stained with PI and DNA content was quantified. Data are presented as mean ± SEM. * *P* < 0.05, ** *P* < 0.01, *** P < 0.001. The *P* values were calculated by two-way analysis of ANOVA for tumor volumes, unpaired Student's *t* test for tumor weights in B and C and unpaired Student's *t* test in G.

To identify previously marketed drugs with potential anti-HCC for drug repurposing, we conducted drug screening assays in three HCC cell lines, namely, Huh7, SNU449, and MHCC-97H, using a library of 546 FDA-approved small-molecule compounds (Table S4). All the drugs were evaluated at a concentration of 10 µM according to the instruction of the library for 24, 48, and 72 hours. Thirty-five compounds, including anthelmintic, antimycotic, antimycotic, antiemetic, antipsychotic, and calcium channel inhibitors, displayed profound cytotoxic effects on HCC cells. Among these effective drugs, oxibendazole and flubendazole showed the most significant antitumor effects in all tested cells (Figure 1A).

To further explore their antitumor effects in HCC, a xenograft nude mouse model was established by subcutaneously injecting Huh7 and MHCC-97H cells. We found that the tumor volumes and weights of subcutaneous xenografted tumors treated with flubendazole were significantly lower than those of tumors treated with the control (Figure 1B and 1C). Furthermore, no significant differences were found in the weights of the mice, livers, or lungs between the two groups (Figure S1A and S1B). H&E staining of liver and lung tissues showed no histopathological damage between the two groups (Figure S1C). Oxibendazole did not affect HCC cell proliferation *in vivo*; however, it exhibited profound cytotoxic effects on HCC cells *in vitro* (Figure S1D).

Figure 1D shows the chemical structure of flubendazole 25. We also tested the cytotoxic effects of flubendazole in a panel of HCC cell lines treated with increasing concentrations, using colony formation assays. The results showed that flubendazole inhibited HCC cell proliferation in a dose-dependent manner (Figure 1E). Similar results were obtained using the CCK-8 kit also, and the concentration that resulted in 50% cell inhibition (IC50; Figure 1F) was determined. Flubendazole displayed a significant antitumor effect at relatively low doses. Among the cell lines, the Huh7 cell line was most sensitive to flubendazole for 72 h treatment (IC50 = 0.263 ± 0.586 µM), whereas SNU449 showed maximum resistance to flubendazole treatment (IC50 = 2.873 ± 0.961 µM). Cell cycle analysis by flow cytometry indicated that cell cycle was arrested at the G2/M phase in the HCC cells treated with 0.25 or 0.5 µM flubendazole (Figure 1G and 1H). Flubendazole has been reported to induce apoptosis in various types of tumors 17. Flow cytometry was used to evaluate the proportion of apoptotic cells stained with annexin V and 7-AAD, and the results showed that flubendazole induced apoptosis in Huh7, MHCC-97H, and SNU449 cells (Figure S2A and S2B).

Taken together, flubendazole effectively inhibits HCC cell proliferation both *in vivo* and *in vitro*.

### Flubendazole downregulates PCSK9 expression in HCC cells

**Figure 2:**
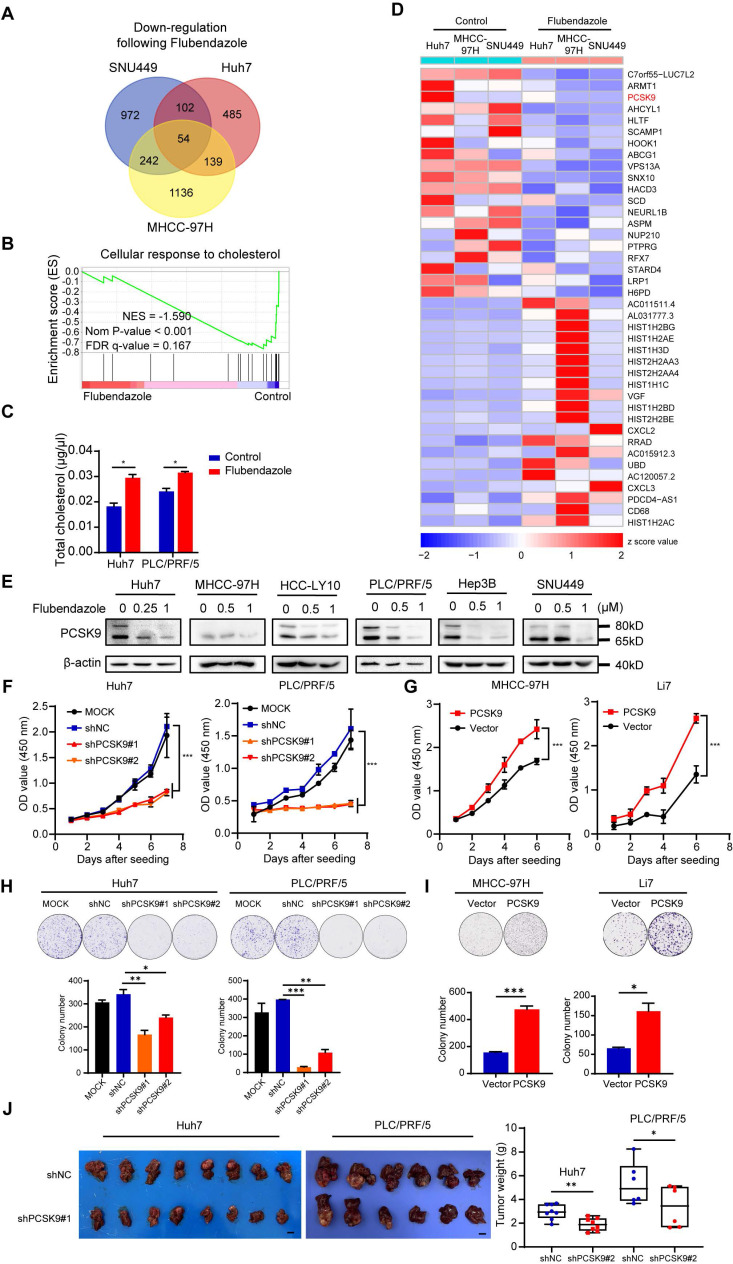
** Flubendazole downregulates PCSK9 expression in HCC cells.** (A) The Venn diagram of downregulated genes in Huh7, MHCC-97H and SNU449 cells after flubendazole treatment compared to control (DMSO). (B) GSEA analysis comparing transcriptomes of HCC cells treated with flubendazole to control (DMSO). (C) Levels of total cholesterol in Huh7 and PLC/PRF/5 cells treated with 0.5 µM flubendazole or control (DMSO). (D) Heatmap of top 20 downregulated and upregulated genes in RNA-sequencing analysis of Huh7, MHCC-97H and SNU449 cells treated with 0.5 µM flubendazole or control (DMSO). PCSK9 are highlighted in red. (E) Western blotting results of PCSK9 expression in Huh7, MHCC-97H, HCC-LY10, PLC/PRF/5, Hep3B and SNU449 cells following 0.5 µM, 1 µM flubendazole treatment or control (DMSO). (F) CCK-8 assays of Huh7 and PLC/PRF/5 cells transfected with MOCK, negative control (shNC) or shPCSK9. (G) CCK-8 assays of MHCC-97H and Li7 cells with overexpression of PCSK9. (H) Upper section shows representative images of colony formation and lower section describes the quantitation histogram of clone numbers in Huh7 and PLC/PRF/5 cells transfected with MOCK, negative control (shNC) or shPCSK9. (I) Upper section shows representative images of colony formation and lower section describes the quantitation histogram of clone numbers in MHCC-97H and Li7 overexpressed with PCSK9 or control vector. (J) Left panel: orthotopic liver tumor from nude mice injected with Huh7 and PLC/PRF/5 cells transfected with shPCSK9#1 or shNC into left hepatic lobe orthotopically after 5 or 8 weeks. Right panel: quantification of xenograft tumor weight (*n* = 8 in Huh7 xenografts; *n* = 6 in PLC/PRF/5 xenografts). Scale bars, 1 cm. Data are presented as mean ± SEM. * *P* < 0.05, ** *P* < 0.01, *** P < 0.001. The *P* values were calculated by unpaired Student's *t* test in C, H, I and J, and two-way analysis of ANOVA in F and G.

To further explore the mechanism underlying the antitumor effects of flubendazole, Huh7, MHCC-97H, and SNU449 cells treated with control or flubendazole were collected for RNA sequencing. Compared with the genes in the control groups, 147 differentially expressed genes (fold change > 1.5) were identified in the three HCC cell lines following flubendazole treatment, of which 54 were downregulated and 93 were upregulated (Figure 2A and S3A). Furthermore, hallmark and GO term enrichment analyses suggested significant downregulation of several pathways, including cellular response to cholesterol, G2/M checkpoint, and mitotic spindle after flubendazole treatment (Figure 2B and S3B). These results confirmed our previous finding that flubendazole induces G2/M arrest in HCC cells.

Cholesterol metabolism has recently attracted the attention of researchers because of its important role in tumor progression and regulation of tumor immunity 26,27. Based on the abovementioned GSEA results, we examined total cholesterol levels in HCC cells treated with control or 0.5 µM flubendazole. The total cholesterol levels were elevated in HCC cells following flubendazole treatment (Figure 2C). Among these differentially expressed genes, PCSK9 (Protein convertase subtilisin/kexin type 9), one of the most significantly downregulated genes in our RNA sequencing results, is of great interest because of its well-known function as a regulator of the degradation of the low-density lipoprotein receptor (LDLR) and its important role in cholesterol metabolism (Figure 2D). We validated the RNA sequencing results by qPCR and western blotting, which confirmed that flubendazole treatment reduced PCSK9 expression in HCC cells in a dose-dependent manner (Figure S3C and 2E).

PCSK9 is associated with the progression of several types of tumors by inhibiting apoptosis and reducing the immune response 17,26. However, the role of PCSK9 in HCC development is controversial 28,29. We first performed lentivirus infection in Huh7 and PLC/PRF/5 cells for the knockdown of PCSK9, whereas performed overexpression of PCSK9 in cell lines Li7 and MHCC-97H with relative low expression of PCSK9 according to their expression in HCC cell lines (Figure S4A and S4B). Efficiency was verified by western blotting (Figure S4C). The CCK-8 assay results indicated that reduced expression of PCSK9 retarded the growth of Huh7 and PLC/PRF/5 cells (Figure 2F). Conversely, PCSK9 overexpression facilitated the proliferation of MHCC-97H and Li7 cells (Figure 2G). In addition, colony formation assays showed that PCSK9 overexpression enhanced clonogenicity in HCC cells (Figure 2H and 2I). To further assess the tumor-forming ability of PCSK9, we established a liver transplantation model by orthotopically injecting Huh7 and PLC/PRF/5 cells transfected with shPCSK9#1 or a negative control (shNC) into the left hepatic lobe. The analysis of average tumor weight showed that reducing PCSK9 expression significantly impaired the tumor formation ability of Huh7 and PLC/PRF/5 cells in nude mice (Figure 2J and S5A).

Moreover, Transwell migration and invasion assays demonstrated that overexpression of PCSK9 promoted the migration and invasion of MHCC-97H and Li7 cells, whereas knockdown of PCSK9 reduced the metastatic ability of Huh7 and PLC/PRF/5 cells (Figure S6A and S6B). H&E staining of the lungs showed that the number of mice with pulmonary metastasis in the shPCSK9#1 group (1 of 8 Huh7 and 0 of 6 PLC/PRF/5 xenografts) was significantly lower than that in the shNC group (6 of 8 Huh7 and 4 of 6 PLC/PRF/5 xenografts), thus verifying the results *in vitro* (Figure S6C).

### PCSK9 is highly expressed in human HCC tissues and predicts poor prognosis

**Figure 3:**
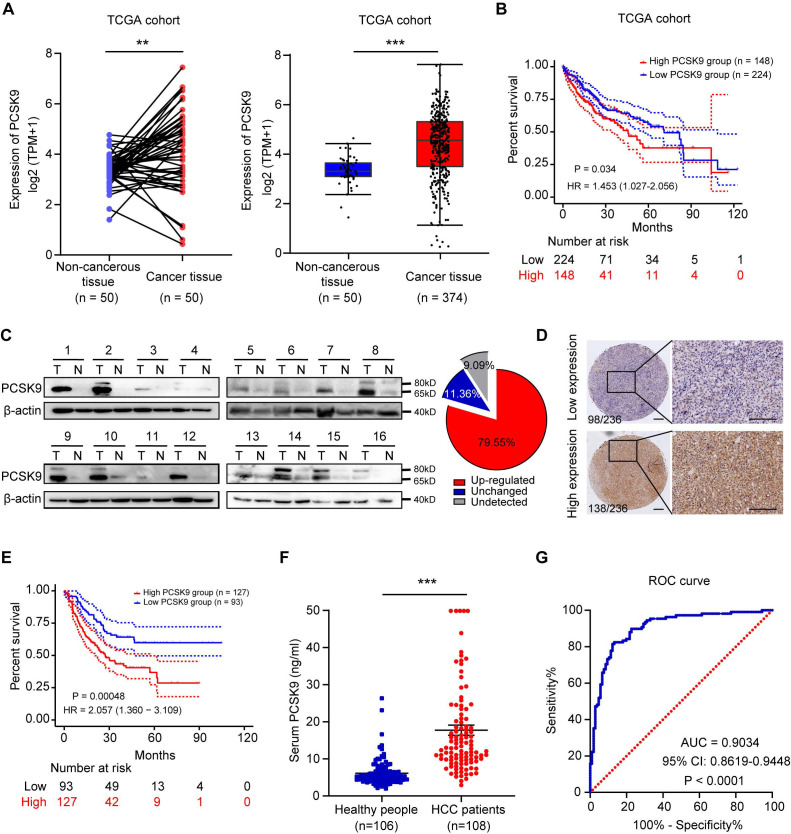
** PCSK9 is highly expressed in human HCC tissues and predicts poor prognosis.** (A) Left panel: the mRNA expression of PCSK9 in 50 paired HCC and para-cancerous tissues in TCGA database. Right panel: the mRNA expression of PCSK9 in 374 HCC and 50 para-cancerous tissues in TCGA database. (B) Kaplan-Meier analysis shows the association of PCSK9 mRNA abundance with overall survival in 372 patients with HCC stratified by mean cut-off value of PCSK9. HR, Hazard Ratio. (C) The protein expression of PCSK9 in 16 paired HCC and adjacent samples in our lab. PCSK9 expressions are identified as upregulated or unchanged in HCC tissues compared with adjacent tissues or undetected. Right pie chart represents the ratio of each category (n = 44). (D) Representative images of IHC staining of PCSK9 expression in 236 HCC tissues samples from our lab. The IHC staining of tumors was divided into low expression group (n = 98) and high expression group (n = 138) according to H&E staining scores. Scale bars, 200 μm. (E) Kaplan-Meier analysis shows the association of PCSK9 protein abundance with overall survival in 220 patients with HCC from our lab. HR, Hazard Ratio. (F) The serum PCSK9 levels of HCC patients (*n* = 108) and healthy people (*n* = 106) samples determined by ELISA. (G) Receiver operating characteristic (ROC) curve analyses of sensitivity and specificity of serum levels of PCSK9 in diagnosis of HCC. AUC, Area Under Curve. Data are presented as mean ± SEM. * *P* < 0.05, ** *P* < 0.01, *** P < 0.001. The *P* values were calculated by paired Student's *t* test in A, unpaired Student's *t* test in F and log-rank test in B and E.

We further explored the correlation between PCSK9 expression and the clinical prognosis of patients with HCC. Pan-cancer analysis of TCGA data showed that PCSK9 is highly expressed in various cancers and is associated with poor prognosis (Figure S7A and S7B). In patients with HCC, PCSK9 expression was significantly higher in HCC samples than that in para-carcinoma tissues (Figure 3A). Moreover, in a TCGA LIHC cohort of 372 patients with HCC stratified by the mean cutoff value, patients with high mRNA expression of PCSK9 in their tumors exhibited shorter overall survival (Figure 3B). Next, we examined the protein expression of PCSK9 in HCC and para-carcinoma tissues using western blotting and found that PCSK9 was upregulated in HCC tissues (Figure 3C and S7C). Furthermore, IHC staining was performed using a human HCC microarray containing 236 HCC tissue samples, which identified 98 patients in the low expression group and 138 patients in the high expression group according to the staining scores (Figure 3D). Consistent with the results from a cohort of patients with HCC collected from our lab, patients with high protein expression of PCSK9 were associated with shorter overall survival (Figure 3E). Notably, on comparing overall survival rates, HCC patients with high PCSK9 expression showed a poor therapeutic response to sorafenib treatment 30 (Figure S7D).

PCSK9 is a secreted protein. Therefore, we performed ELISA to detect PCSK9 levels in the serum samples of 108 patients with HCC and 106 healthy individuals. As shown in Figure 3F, the serum PCSK9 levels in patients with HCC were significantly higher than those in the healthy group. The diagnostic value of PCSK9 was evaluated using the receiver operating characteristic (ROC) curve, which demonstrated that serum PCSK9 may assist in diagnosing HCC with an AUC of 0.9034 (95% CI, 0.8619-0.9448) (*P* < 0.0001) (Figure 3G). The demographic information of HCC patients in ELISA results is listed in Table S5.

Taken together, these results suggest that high expression of PCSK9 in patients with HCC correlates with poor overall survival and that serum PCSK9 levels may be a promising diagnostic biomarker for HCC.

### Flubendazole suppresses tumorigenicity by targeting PCSK9 directly in HCC

**Figure 4:**
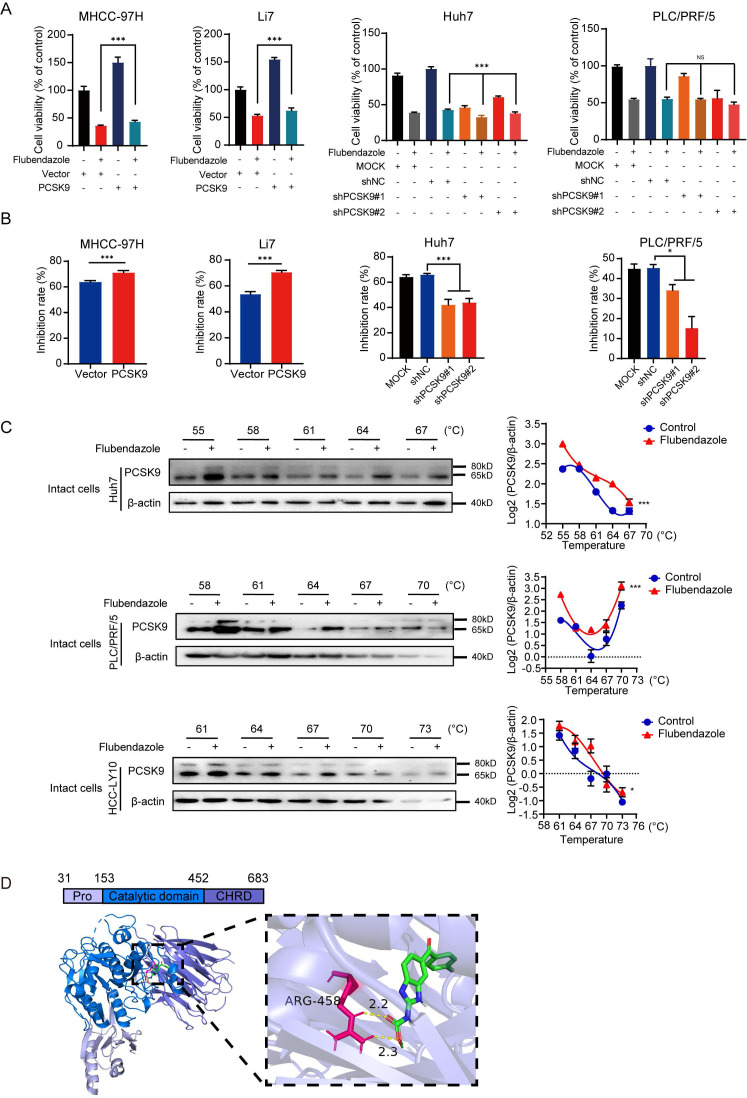
**Flubendazole suppresses tumorigenicity via targeting PCSK9 directly in HCC.** (A) Cell viabilities of MHCC-97H and Li7 cells with overexpression of PCSK9 and Huh7 and PLC/PRF/5 with knockdown of PCSK9 treated with flubendazole for 72 hours. (B) Inhibition rates of MHCC-97H and Li7 cells with overexpression of PCSK9 and Huh7 and PLC/PRF/5 with knockdown of PCSK9 treated with flubendazole for 72 hours. (C) Left panel shows thermal shift analysis of Huh7, PLC/PRF/5 and HCC-LY10 cells treated with flubendazole or DMSO. Levels of PCSK9 are normalized relative to amounts of β-actin and are shown in the right panel. (D) Left panel shows the binding model of PCSK9 (blue cartoon) combined with flubendazole (green sticks) with the lowest binding free energy. Right panel indicates the zoom binding amino acid of PCSK9 (red sticks) and hydrogen bond with the distance values (yellow sticks). Pro, subtilisin-like pro domain of PCSK9; CHRD, C-terminal domain of PCSK9. Data are presented as mean ± SEM. * *P* < 0.05, ** *P* < 0.01, *** P < 0.001. The *P* values were calculated by two-way Analysis of ANOVA in C, unpaired Student's *t* test in A and B.

These results demonstrated the tumorigenicity of PCSK9 in HCC. Interestingly, the sensitivity of HCC cells to flubendazole appeared to be correlated with the expression of PCSK9 (Figure 1F, S4A, and S4B). To confirm the tumorigenic ability of PCSK9, we hypothesized that flubendazole may inhibit tumor growth in a PCSK9 dependent manner. PCSK9-knockdown Huh7 and PLC/PRF/5 cells were treated with flubendazole. The CCK8 assay results showed that PCSK9 knockdown rendered HCC cells insensitive to flubendazole treatment, whereas PCSK9 overexpression augmented the sensitivity of HCC cells to flubendazole treatment (Figure 4A and 4B).

To further determine the mechanism by which flubendazole regulates PCSK9 protein, we performed CETSA using intact Huh7, PLC/PRF/5, and HCC-LY10 cells with flubendazole or control treatment. Western blotting results showed a shift in the denaturation temperature of PCSK9 in cells treated with flubendazole compared with that in the control group (Figure 4C), which indicated that flubendazole could bind to PCSK9 directly. Moreover, using molecular docking analysis, we determined the potential binding mode of PCSK9 with flubendazole, with a lowest binding affinity of -9.4 kcal/mol. Flubendazole was predicted to form a hydrogen bond with the ARG458 residue in the C-terminal domain of PCSK9, which plays a vital role in PCSK9 internalization (Figure 4D) 31.

Together, flubendazole suppresses proliferation of HCC cells via targeting PCSK9 directly.

### Flubendazole downregulates the Hedgehog signaling pathway by inhibiting PCSK9

**Figure 5:**
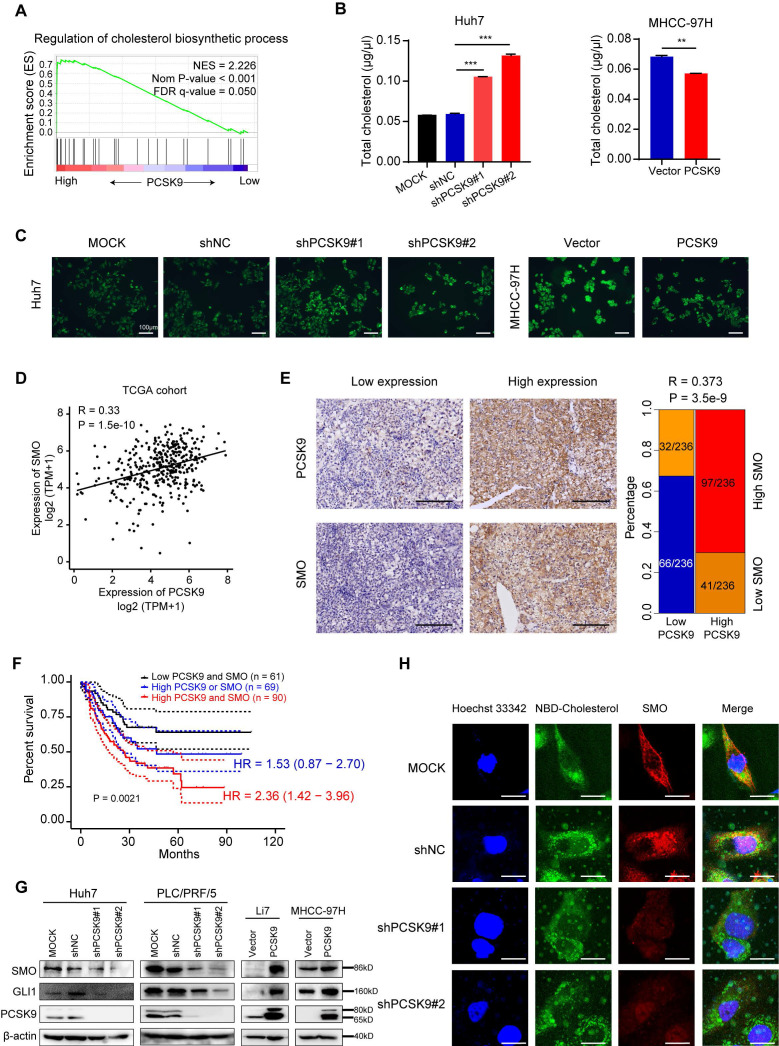
** PCSK9 upregulates Hedgehog pathway via regulating cholesterol levels.** (A) GSEA analysis performed in a TCGA cohort of 374 HCC tissues stratified by mean cut-off value of PCSK9 expression. The data identified pathways of regulation of cholesterol biosynthetic process. NSE, normalized enrichment score. (B) Total cholesterol levels in Huh7 cells with knockdown of PCSK9 and MHCC-97H cells with overexpression of PCSK9. (C) Fluorescence images of Huh7 cells with knockdown of PCSK9 and MHCC-97H cells with overexpression of PCSK9 incubated with NBD cholesterol for 1 hour. Scale bars, 100 μm. (D) Correlation of mRNA expression of SMO and PCSK9 in TCGA database (Spearman correlation coefficient R = 0.33, *P* < 0.001). (E) Left panel shows the representative images of IHC staining of concurrent high or low PCSK9/SMO expression in 236 HCC tissues samples. Blue square presents the number of patients with both low expression of PCSK9 and SMO. Red square presents the number of patients with concurrent high expression of PCSK9 and SMO (R = 0.373, *P* < 0.001) Scale bars, 200 μm. (F) Kaplan-Meier survival analysis of 220 HCC patients with high expression of SMO or/and PCSK9 from our lab. HR, Hazard Ratio. (G) Western blotting of protein expressions of SMO, Gli1 and PCSK9 in HCC cells with overexpression or knockdown of PCSK9. (H) Immunofluorescence images of Huh7 cells with knockdown of PCSK9 or control stained with NBD-Cholesterol (Green), SMO (Red) and Hoechst 33342 (Blue). Scale bars, 20 μm. Data are presented as mean ± SEM. * *P* < 0.05, ** *P* < 0.01, *** P < 0.001. The *P* values were calculated by unpaired Student's *t* test in B, Spearman correlation coefficient in D and log-rank test in F.

To further explore the molecular mechanism by which PCSK9 promotes HCC progression, we performed GSEA of a TCGA cohort of 374 HCC tissues stratified by the mean cut-off value of PCSK9 expression. Several pathways were identified, including the regulation of the cholesterol biosynthetic process, DNA replication, G2/M checkpoint, mitotic sister chromatid segregation, and DNA replication enrichment with high PCSK9 expression (Figure 5A and S8A). Consistent with this analysis, we performed cell cycle analysis, and the results showed that knockdown of PCSK9 increased the portion of G2/M phase in the Huh7 cells while overexpression of PCSK9 induced a marked reduction in the G2/M phase in MHCC-97H and Li7 cells (Figure S8B). These results were consistent with the findings in HCC cells treated with flubendazole.

We further confirmed that the knockdown of PCSK9 resulted in an increase in total cholesterol levels in Huh7 cells, and overexpression of PCSK9 decreased total cholesterol levels in MHCC-97H cells (Figure 5B). Incubation of HCC cells with NBD-cholesterol also showed that PCSK9 knockdown enhanced the uptake of cholesterol into cells, whereas PCSK9 overexpression inhibited the internalization of cholesterol into cells (Figure 5C). The Hh signaling pathway is correlated with cell proliferation and differentiation during embryonic development 32,33. Disinhibition of SMO is a necessary step for activation of the Hh signaling pathway, and cholesterol modification is required for SMO activation 34,35. Recent data have shown that high expression and activation of the Sonic Hedgehog (Shh) signaling pathway are associated with HCC progression and poor prognosis 36. Therefore, we hypothesized that PCSK9 regulates the Hh signaling pathway by regulating cholesterol levels.

To validate our hypothesis, we analyzed the correlation between PCSK9 and Hh signaling pathways in TCGA database. The mRNA levels of PCSK9 positively correlated with SMO (R = 0.33, *P* < 0.001) and Gli1 (R = 0.23, *P* < 0.001) expression in HCC tissues (Figure 5D and S8C). A positive correlation between PCSK9 and SMO protein expression was confirmed by the IHC analysis of 236 HCC samples obtained from our laboratory (Figure 5E). Kaplan-Meier survival analysis showed that HCC patients with high expression of SMO exhibited shorter overall survival (Figure S8D), and patients with high expression of both PCSK9 and SMO demonstrated the worst overall survival (Figure 5F). Intriguingly, PCSK9 knockdown decreased the protein expression levels of SMO and Gli1 in Huh7 and PLC/PRF/5 cells, whereas PCSK9 overexpression increased the expression levels of SMO and Gli1 in MHCC-97H and Li7 cells (Figure 5G). Activation of SMO involves the modification of cholesterol with SMO protein at the cell membrane 34. Therefore, we performed immunofluorescence experiments and found decreased expression of SMO and reduced co-localization of SMO and cholesterol at the cell membrane in Huh7 cells with PCSK9 knockdown compared with those in the control cells (Figure 5H). These results indicate that PCSK9 activates SMO in the Hh pathway through cholesterol accumulation at the cell membrane.

**Figure 6:**
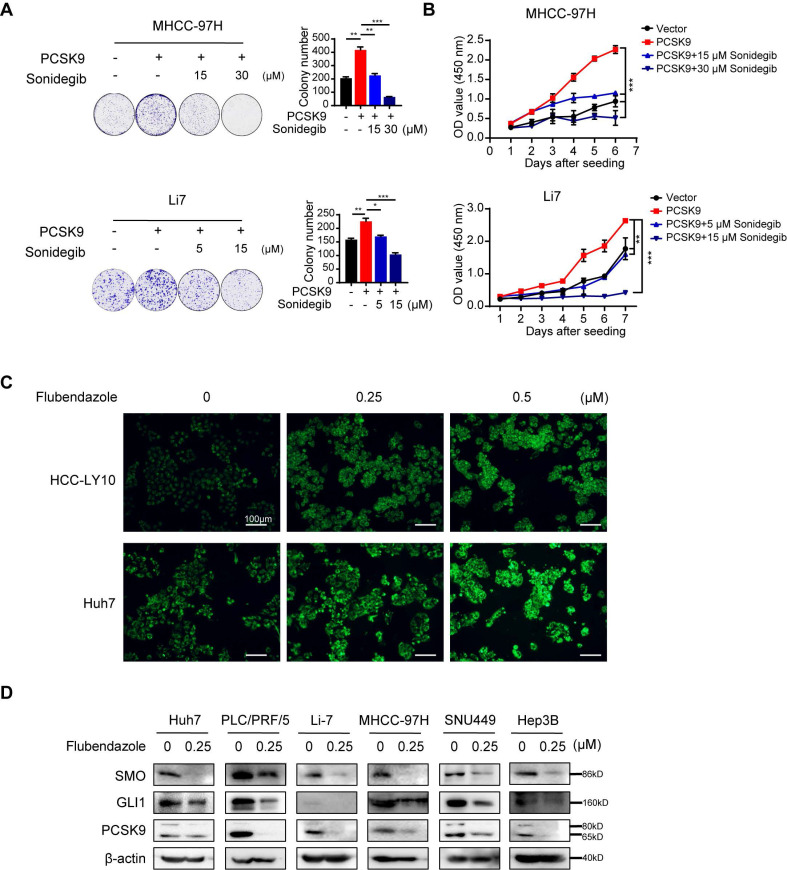
** Flubendazole downregulates the Hh signaling pathway by targeting PCSK9.** (A) Representative images of colony formation and quantification histogram of clone numbers in MHCC-97H and Li7 cells overexpressing PCSK9 or control vector treated with SMO inhibitor, sonidegib (5, 15 or 30 µM). (B) CCK-8 assays examined the cell viability of HCC cells transfected with PCSK9 overexpression or control vector treated with SMO inhibitor, sonidegib (5, 15 or 30 µM). (C) Fluorescence images of HCC cells treated with control (DMSO), 0.25 μM and 1 μM flubendazole and then incubated with NBD cholesterol for 1 hour. Scale bars, 100 μm. (D) The protein expressions of SMO, Gli1 and PCSK9 in HCC cells treated with 0.25 µM flubendazole or control. Data are presented as mean ± SEM. * *P* < 0.05, ** *P* < 0.01, *** P < 0.001. The *P* values were calculated by unpaired Student's *t* test in A, two-way Analysis of ANOVA in B.

Furthermore, treatment with the SMO inhibitor sonidegib, in MHCC-97H and Li7 cells with PCSK9 overexpression or vector control showed that soniedegib inhibited the ability of cell proliferation and colony formation induced by PCSK9 (Figure 6A and B). These results indicate that PCSK9 promotes HCC progression via the activation of SMO in the Hh signaling pathway.

As described above, flubendazole reduced PCSK9 expression in HCC cell lines and affected total cholesterol levels (Figure 2C). NBD-cholesterol staining also showed an increase in cholesterol uptake by HCC cells after flubendazole treatment (Figure 6C and S8E). Here, we also observed that SMO and Gli1 in the Hh signaling pathway were downregulated in HCC cells treated with flubendazole compared with those in the control group (Figure 6D).

Taken together, flubendazole increases the cellular uptake of cholesterol in HCC cells by inhibiting PCSK9, which results in suppressed cholesterol levels modified with SMO and leads to the inhibition of the Hh signaling pathway.

### Combination of flubendazole and lenvatinib elicits synergic effect in HCC treatment

**Figure 7:**
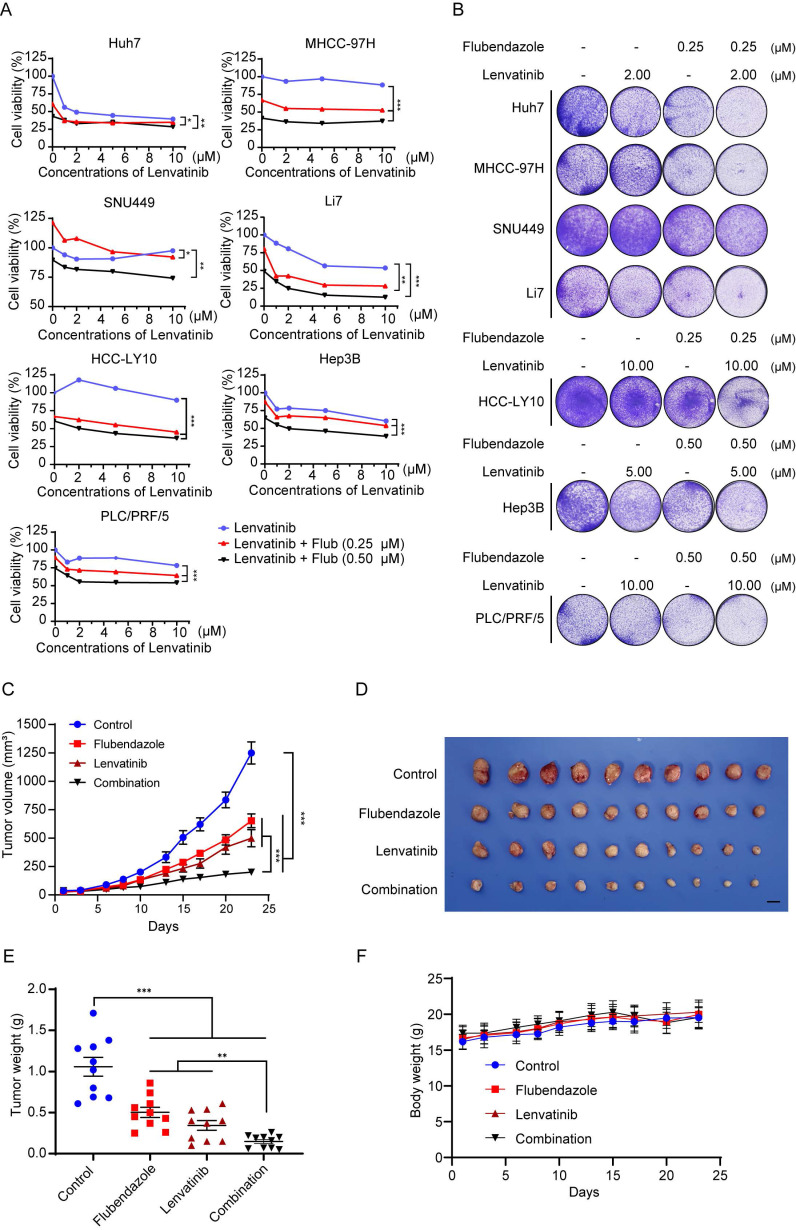
** Combination of flubendazole and lenvatinib elicits synergic effect in HCC treatment.** (A) Cell viability of HCC cells treated with lenvatinib alone or combined with 0.25 µM or 0.50 µM flubendazole. (B) Clonogenicity of HCC cells treated with lenvatinib, flubendazole or in combination therapy. (C) Subcutaneous tumor volumes of MHCC-97H (*n* = 10) in nude mice treated with lenvatinib (4 mg/kg) alone or flubendazole (40 mg/kg) alone or combination of two drugs by peritoneal injection. (D) Image of tumors in nude mice treated with lenvatinib (4 mg/kg) alone or flubendazole (40 mg/kg) alone or combination of two drugs by peritoneal injection. (E) and (F) Tumor weights and body weights of nude mice for the treatment as described above. Data are presented as mean ± SEM. * *P* < 0.05, ** *P* < 0.01, *** P < 0.001. The *P* values were calculated by two-way analysis of ANOVA in A, C, and F and by unpaired Student's *t* test in E.

According to the Kaplan-Meier curve, high expression of PCSK9 is correlated with poor outcomes with sorafenib, which is the first-line drug for the treatment of HCC (Figure S7D). We explored whether flubendazole could enhance the cytotoxic effect of lenvatinib, another multiple kinase inhibitor, in HCC cells. Lenvatinib exhibited dose-dependent cytotoxic effects on HCC cell lines. We combined different concentrations of lenvatinib with 0.25 M or 0.5 µM flubendazole to treat a panel of HCC cell lines. The CCK-8 assay results showed that the combination of the two drugs displayed a stronger antitumor effect in HCC cells (Figure 7A). The CDI was calculated to quantify the combined effects of flubendazole and lenvatinib. The combined treatment showed a synergistic effect in all HCC cell lines, except Hep3B, compared with the theoretical additive effect (Table S6). Although the combination treatment in Hep3B cells did not show a significant synergistic effect, an additive effect was confirmed. Colony formation assays showed that flubendazole enhanced the cytotoxic effects of lenvatinib (Figure 7B).

Furthermore, we confirmed the synergistic effects of flubendazole and lenvatinib in a xenograft nude mouse model. Our results showed that the tumor volumes and weights were significantly lower in the drug combination group than in the other groups (Figure 7C-E and S9A). No differences were found in the body weights of the mice in any of the groups (Figure 7F).

**Figure 8:**
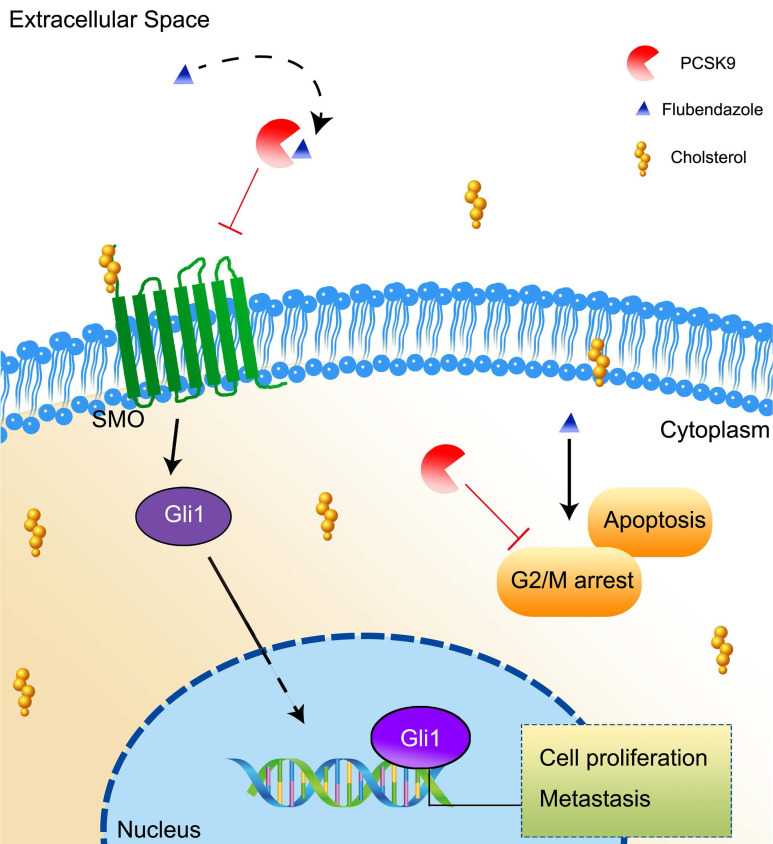
A schema showing that flubendazole inhibits HCC proliferation by targeting PCSK9 directly and results in an increasing uptake of cholesterol in the HCC cells.

In summary, our study found that flubendazole suppressed HCC growth in a PCSK9-dependent manner via a direct interaction. The inhibition of PCSK9 results in an increase in the uptake of cholesterol by HCC cells and inhibits the activation of the downstream molecules SMO and Gli1 in the Hh signaling pathway. PCSK9 is overexpressed in HCC and promotes HCC progression by reducing apoptosis and arresting transduction to G2/M in cell cycle (Figure 8).

## Discussion

HCC is one of the most common tumors with a poor prognosis and high mortality worldwide. Due to the difficulties in early diagnosing and the rapid progress of HCC, patients with HCC are usually diagnosed with advanced-stage disease that is challenged by lack of effective therapy 37. Therefore, there is an urgent need for novel effective drugs and molecular targets. Recently, drug repurposing has become a popular method of drug discovery with the advantages of clear pharmacokinetics, pharmacodynamics, side effects, and dosing regimens 38. In our study, we screened 546 drugs in an FDA-approved drug library and, for the first time, identified flubendazole as an anti-HCC drug that exerted the most potent anti-tumor potential both *in vitro* and *in vivo*.

Flubendazole is an FDA-approved anthelmintic drug used in human parasite therapy 11. Recent studies have shown that flubendazole also plays an anti-neoplastic role in different kinds of cancers, such as breast cancer, leukemia, myeloma, glioblastoma, prostate cancer, colon cancer, oral squamous carcinoma, and non-small cell lung cancer 12,13,15,17,39-42. In this study, we investigated the antitumor effects of flubendazole in HCC cells* in vitro* and *in vivo*. Similar to that in previous studies on other cancers 17, we verified the induction of apoptosis and G2/M cell cycle arrest in HCC cell lines treated with flubendazole 43. Our findings provide clues for the development of new drug candidates for HCC chemotherapy.

In addition to its reported function in cancer, we discovered that flubendazole can inhibit the expression of PCSK9 via direct interactions. PCSK9 is a member of the pro-protein convertase family, and its function in cholesterol metabolism was initially determined in hypercholesterolemia caused by a PCSK9 mutation 44,45. Further mechanistic investigation indicated that PCSK9 regulates the degradation of the LDLR by inducing its translocation to lysosomes and endosomes, leading to an increase in low-density lipoprotein cholesterol (LDL-C) levels 46. In our study, we predicted that flubendazole interacts with the C-domain of PCSK9, which may offer new clues for developing PCSK9 inhibitors. Cholesterol metabolism has been reported as a source of energy supply for tumor cells and regulator of the tumor microenvironment that reduces tumor immunity 27,47-49. Recently, PCSK9 was found to promote tumor progression by regulating apoptosis, metastasis, and tumor immunity via lysosomal degradation of MHC Class I molecules 26,29,50,51. Increased levels of PCSK9 have been observed in patients with HCC and infected with chronic HCV 52.

However, decreased PCSK9 expression has been reported 53, and reports on the function of PCSK9 in HCC are controversial 28,29. Based on the analysis of TCGA data and HCC tissues in our laboratory, we found that PCSK9 was overexpressed in HCC tissues, and high expression of PCSK9 predicted poor survival. PCSK9 is a secreted protein, and we determined the diagnostic value of serum PCSK9 with high sensitivity and specificity using ROC analysis. Further studies need to be conducted with larger sample sizes and in patients with hepatitis and liver cirrhosis.

The Hh signaling pathway is an ancient and traditional signaling pathway that plays a vital role in embryonic development and primary cilium maturation 32,54. Aberrant activation of the Hh signaling pathway leads to tumorigenesis and metastasis via activation of the caspase pathway 33,36. The Hh pathway cascade includes the binding of secreted Hh proteins to Ptc homolog proteins, which leads to the relief of its inhibition on the seven-pass transmembrane protein smoothened (SMO) 35,55,56. SMO is an important molecule in the Hh pathway that is highly expressed in many types of cancers, including HCC, and is associated with poor prognosis and good cancer progression 57,58. Activation of SMO improves the downstream transcriptional activators of the glioma-associated oncogene family, including Gli1, Gli2, and Gli3 59. Aberrant activation of the Hh pathway, especially the Hh and SMO proteins, has been reported to be associated with cancer progression and metastasis in HCC 33,57,58,60,61. It has been reported that the activation of Hh and SMO in the Hh pathway is modified by cholesterol 62,63 and that cholesterol can serve as a second messenger to communicate with and activate SMO 34. The inhibition of cholesterol biosynthesis by veratrum alkaloids leads to teratogenesis during pregnancy by inhibiting Hh signaling 64. Overexpression of PCSK9 inhibits the uptake of LDL-C into cells through LDLR, thus increasing the amount of extracellular cholesterol in the blood 65. Dysregulation of cholesterol activates SMO and the Hh signaling pathway cascade 58. Here, we found that knockdown of PCSK9 elevated the level of total cholesterol in HCC cells, whereas overexpression of PCSK9 displayed the opposite effects. Furthermore, the knockdown of PCSK9 decreases the expression of SMO, and its colocalization with cholesterol at the cell membrane leads to the inhibition of tumor progression. Treatment with an SMO-specific inhibitor abrogated the proliferative effects of PCSK9. These data support the hypothesis that PCSK9 activates Hh signaling by regulating cholesterol uptake in HCC cells. Consistently, flubendazole treatment elevated total cholesterol levels in HCC cells by downregulating PCSK9, SMO, and Gli1 expression. Interestingly, mebendazole, an anthelmintic agent and benzimidazole drug, has been reported to serve as a potential Hh inhibitor 66.

Sorafenib and lenvatinib are multiple kinase inhibitors and first-line drugs used to treat patients with advanced HCC 67. The Kaplan-Meier curve indicated that high PCSK9 expression correlates with poor response to sorafenib in patients with HCC. According to Sun* et al*., the suppression of S-palmitoylation by PCSK9 sensitized sorafenib therapy in HCC 68. The anthelmintic agent mebendazole augments the sensitivity of HCC cells to sorafenib by targeting BCL2 and MAPK 69. In our study, we demonstrated that flubendazole can inhibit the Hh pathway by inhibiting PCSK9. The Hh pathway is a classic pathway that plays a crucial role in hepatocellular progression and induces the MAPK/ERK pathway, which plays an important role in lenvatinib resistance 70. Hu *et al*. showed that lenvatinib drug resistance was associated with aberrant cholesterol metabolism and lipid raft activation 71. Furthermore, Hu *et al*. showed that inhibiting the Hh pathway in CD133-positive hepatocellular carcinoma can improve the outcomes of lenvatinib therapy 72. Based on the above research, we hypothesized that flubendazole could overcome the drug resistance of lenvatinib by inhibiting the Hh pathway and downstream molecules. Here, we determined the synergistic effect of a combination of flubendazole and lenvatinib, which provides directions and a basis for clinical experiments and personalized treatment. Further clinical trials are needed to explore the effects of flubendazole in HCC treatment.

In conclusion, we validated the antitumor effects of flubendazole in HCC for the first time. PCSK9, a target of flubendazole, is overexpressed in HCC tissues and predicts poor survival. PCSK9 upregulates the expression of SMO and Gli1 in the Hh signaling pathway to promote HCC growth and metastasis. Flubendazole inhibits HCC proliferation by targeting PCSK9 via the Hh pathway. Furthermore, flubendazole enhances the antitumor effects of lenvatinib in HCC treatment. These results provide novel insights into the effects of flubendazole and clues to the mechanism of new targets in HCC treatment.

## Supplementary Material

Supplementary figures and tables 1-3, 5, 6.

Supplementary table 4.
