# Supplementary material for: Drug Repurposing Flubendazole to Suppress Tumorigenicity via PCSK9-dependent Inhibition and Potentiate Lenvatinib Therapy for Hepatocellular Carcinoma
Source: Int J Biol Sci. 2023 Apr 23;19(7):2270–88. doi: 10.7150/ijbs.81415 (PMC10158015; doi:10.7150/ijbs.81415)
Supplement: Supplementary file 1 — Supplementary figures and tables 1-3, 5, 6. [file ijbsv19p2270s1.pdf]

Supplementary Figures and legends

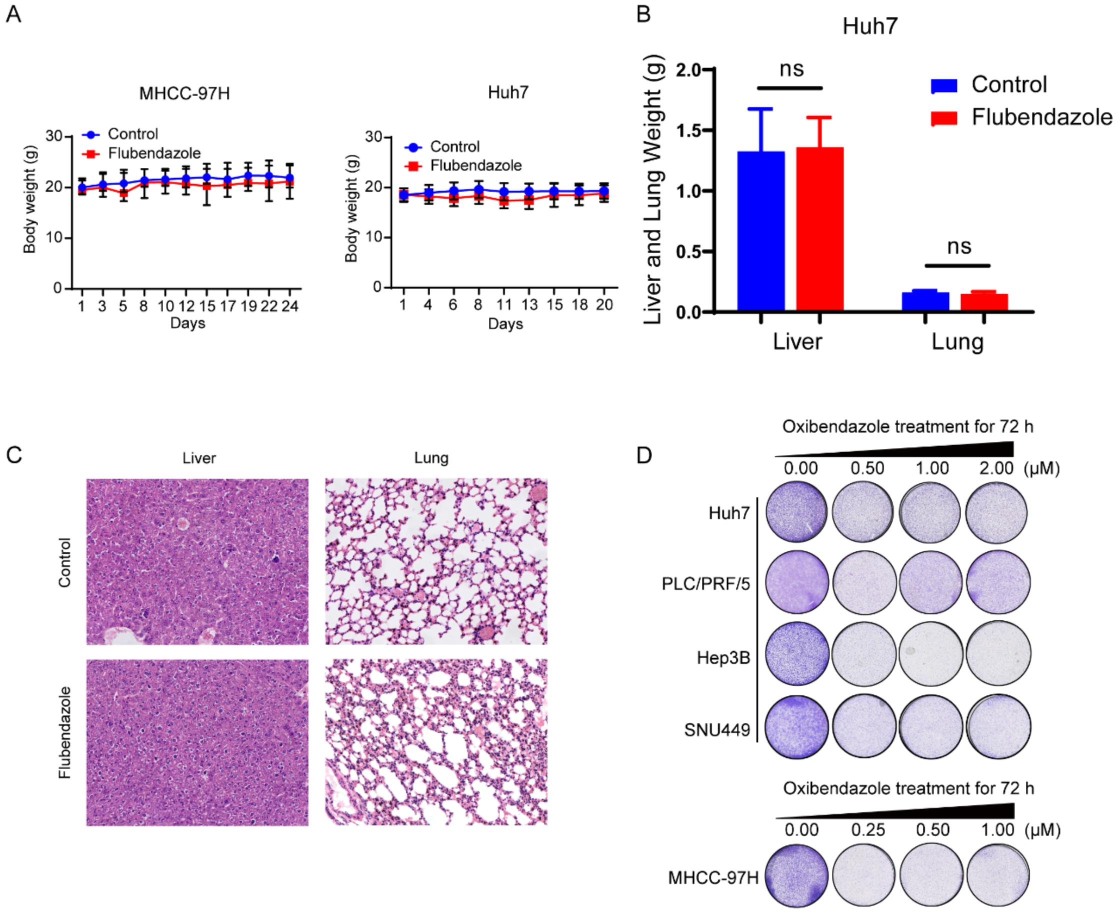

Figure S1. The toxic effects of flubendazole and oxibendazole inhibiting HCC proliferation *in vitro*.

A Body weights of nude mice during control (DMSO) or flubendazole treatment.

B Liver and lung weights of nude mice treated with flubendazole or control (DMSO).

C Representative images of H&E stained livers and lungs of mice treated with flubendazole or control.

D Colony formation assay of HCC cells treated with 0.50, 1.00 and 2.00 μM oxibendazole for 72 hours.

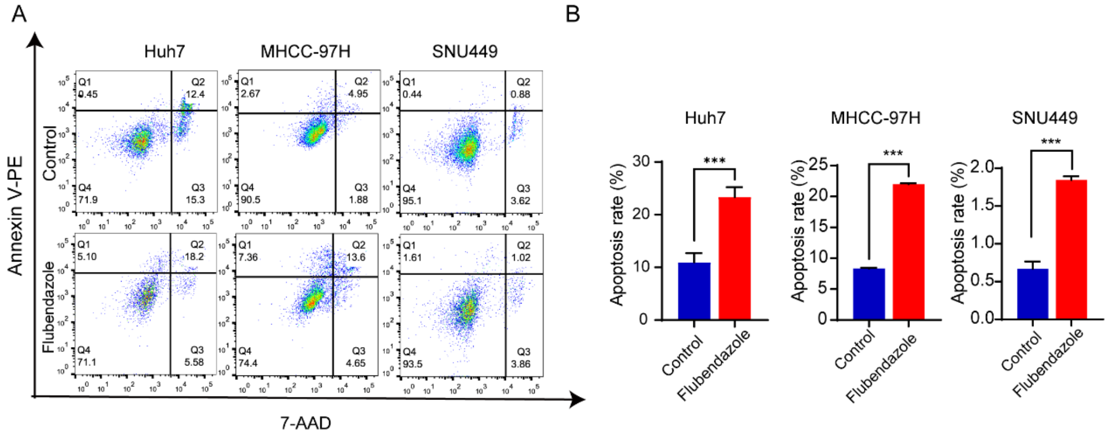

Figure S2. Flubendazole promotes apoptosis of HCC cells. Apoptotic rate of cells treated with control (DMSO) or 0.25 or 0.5μM flubendazole for 72 hours was conducted by flow cytometry analysis of Annexin-V/7-AAD staining. Representative images (**A**) and quantification of apoptotic cells (**B**) are shown. $P < 0.05$, ** $P < 0.01$, *** $P < 0.001$. The P values were calculated by unpaired Student's t test **B**. The data are presented as mean ± SEM.

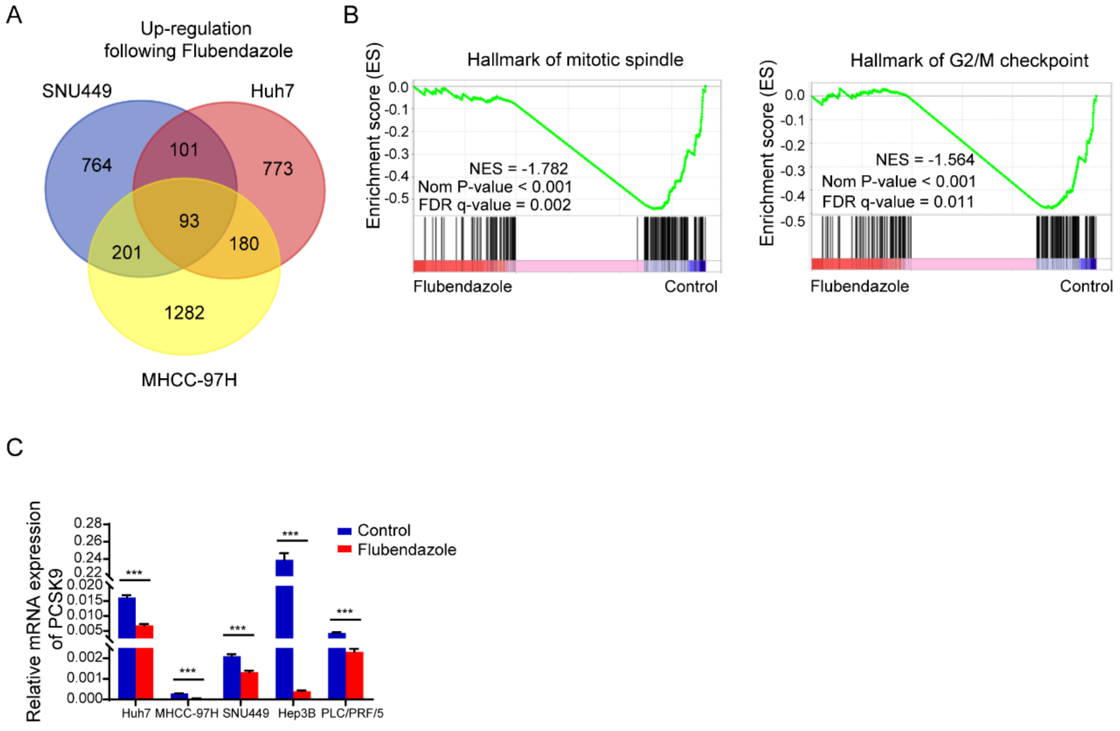

Figure S3. Flubendazole reduces the expression of PCSK9 in HCC cell lines.

A Venn diagram of upregulated genes in Huh7, MHCC-97H and SNU449 cells after flubendazole treatment compared to controls. **B** GSEA analysis comparing transcriptomes of HCC cells treated with flubendazole to control (DMSO). NSE, normalized enrichment score. **C** The mRNA expression of PCSK9 in HCC cell lines treated with or without 0.5 μM flubendazole determined by qPCR.

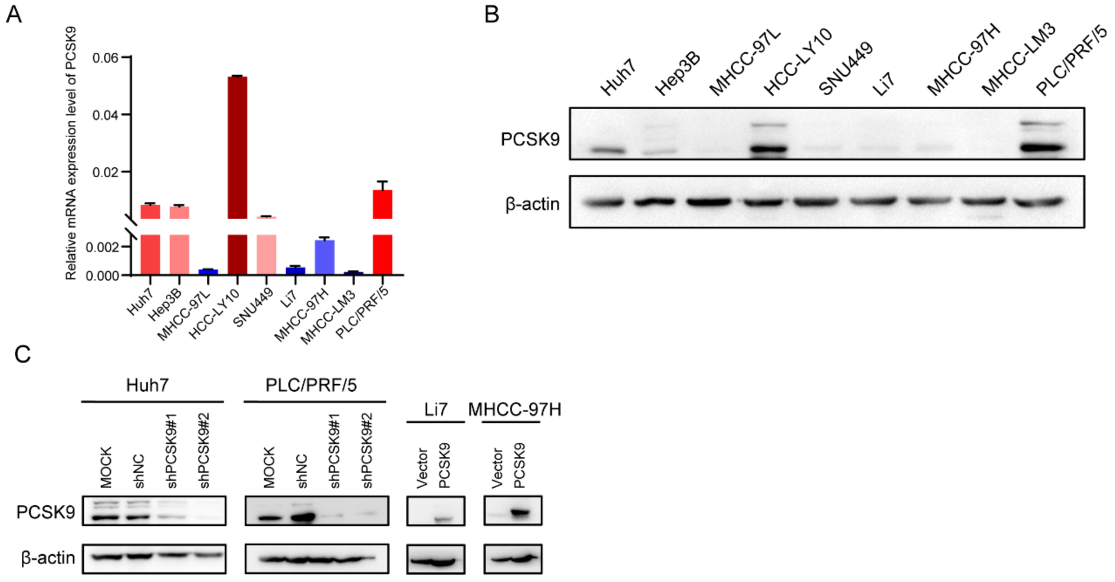

Figure S4. The mRNA and protein expressions of PCSK9.

A Relative mRNA expression of PCSK9 in different HCC cell lines. **B** Western blotting analysis of protein expression levels of PCSK9 in different HCC cell lines. **C** Efficiencies of overexpression and knockdown of PCSK9 verified by western blotting.

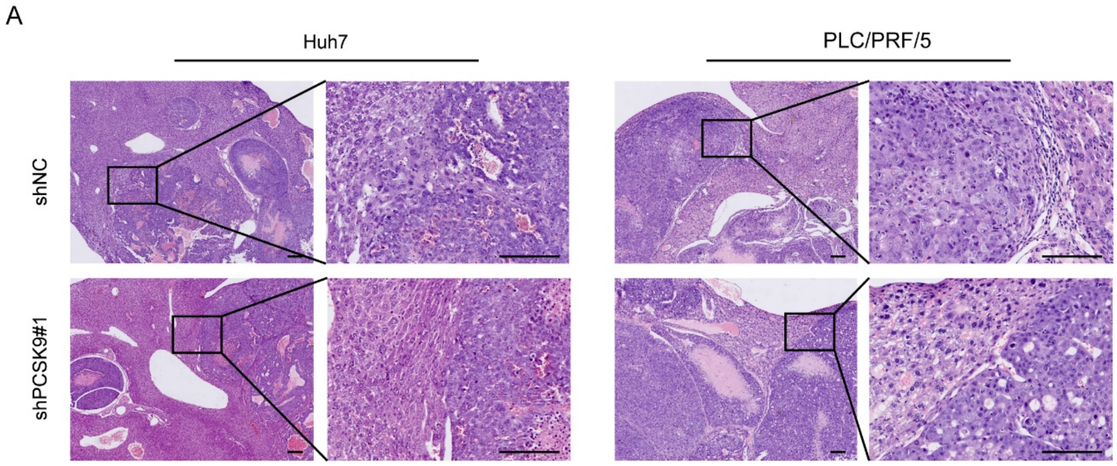

Figure S5. PCSK9 promotes proliferation and metastasis of HCC cells *in vivo*.

A Representative images of H&E stained orthotopic liver tumor. Scale bars, 200 μm.

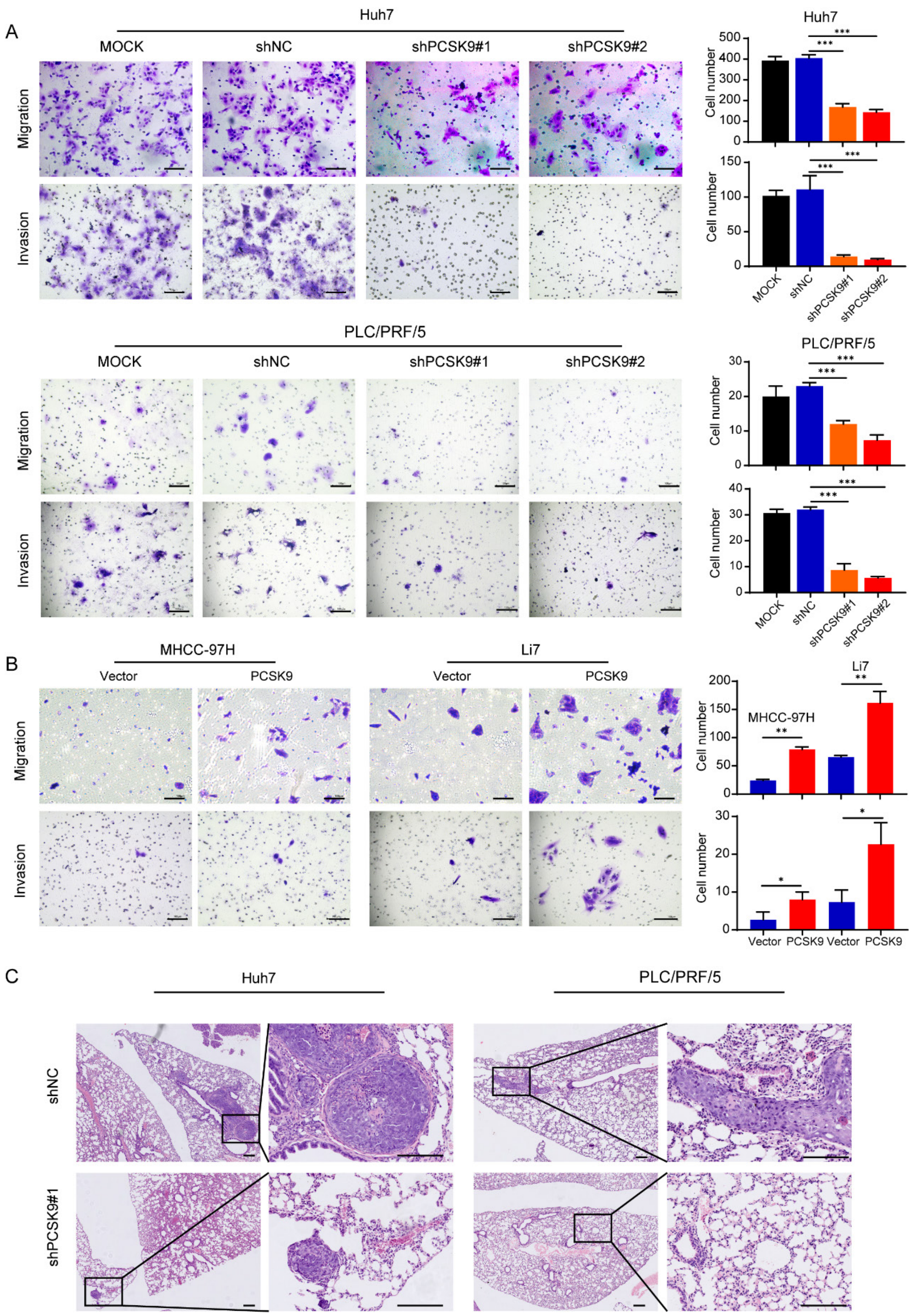

Figure S6. PCSK9 promotes metastasis of HCC cells *in vitro* and *in vivo*.

A-B Representative images of transwell migration and invasion assay of Huh7 and PLC/PRF/5 cells following knockdown of PCSK9 and Li7 and MHCC-97H cells following overexpression of PCSK9. Scale bars, 100 μm

C Representative images of H&E stained lung section with metastatic nodules. Scale bars, 200 μm.

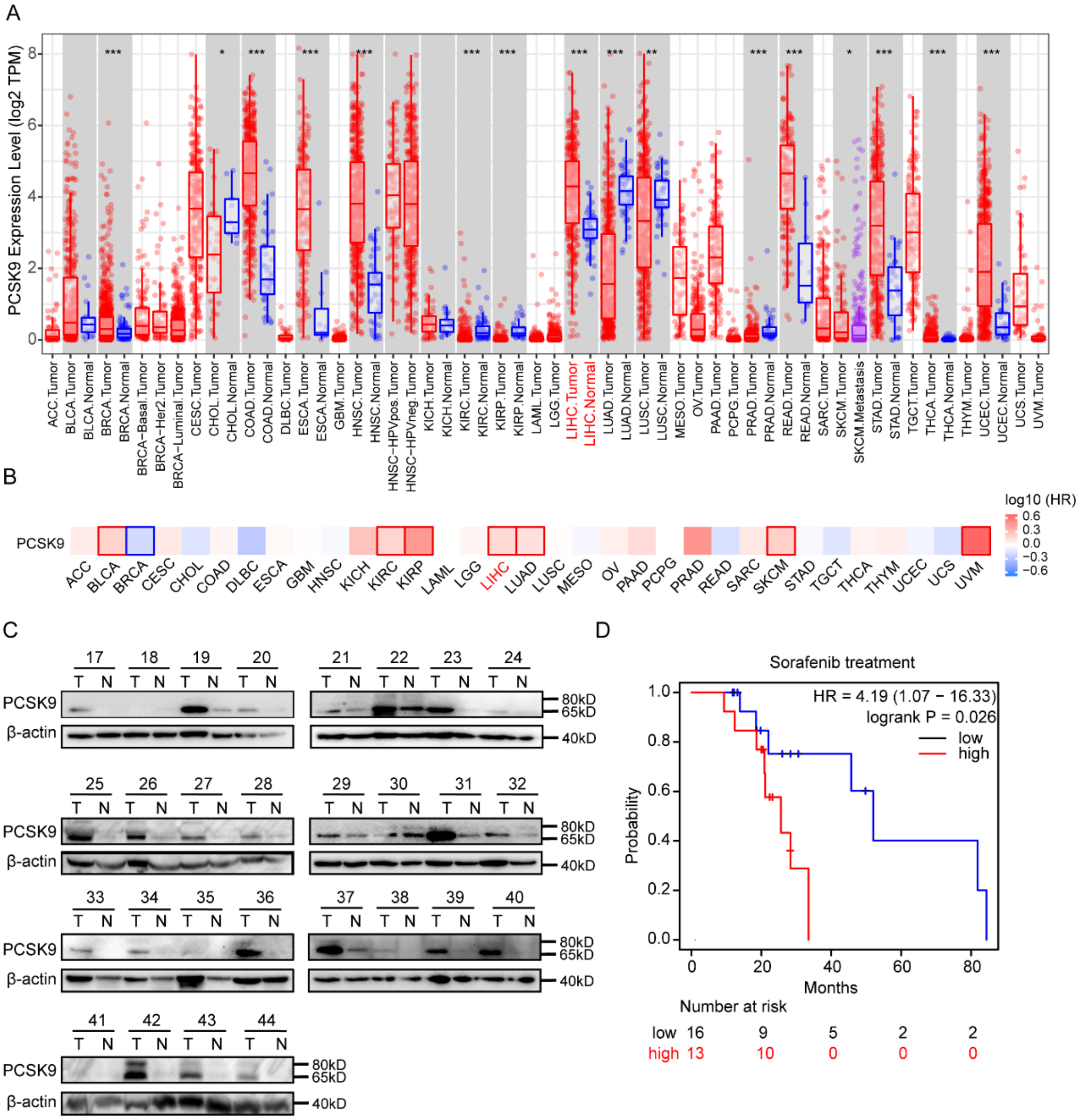

Figure S7. PCSK9 is overexpressed in different kinds of cancers and associated with poor prognosis.

A The mRNA expression levels of PCSK9 in diverse kinds of cancers according to TCGA database. **B** Hazard Ratio of patients with high expression of PCSK9 in various kinds of cancers in TCGA database. HR, Hazard Ratio. **C** Protein expression levels of paired HCC and adjacent samples in our labs. **D** The overall survival rates of HCC patients with low or high PCSK9 expression responding to Sorafenib treatment in TCGA database.

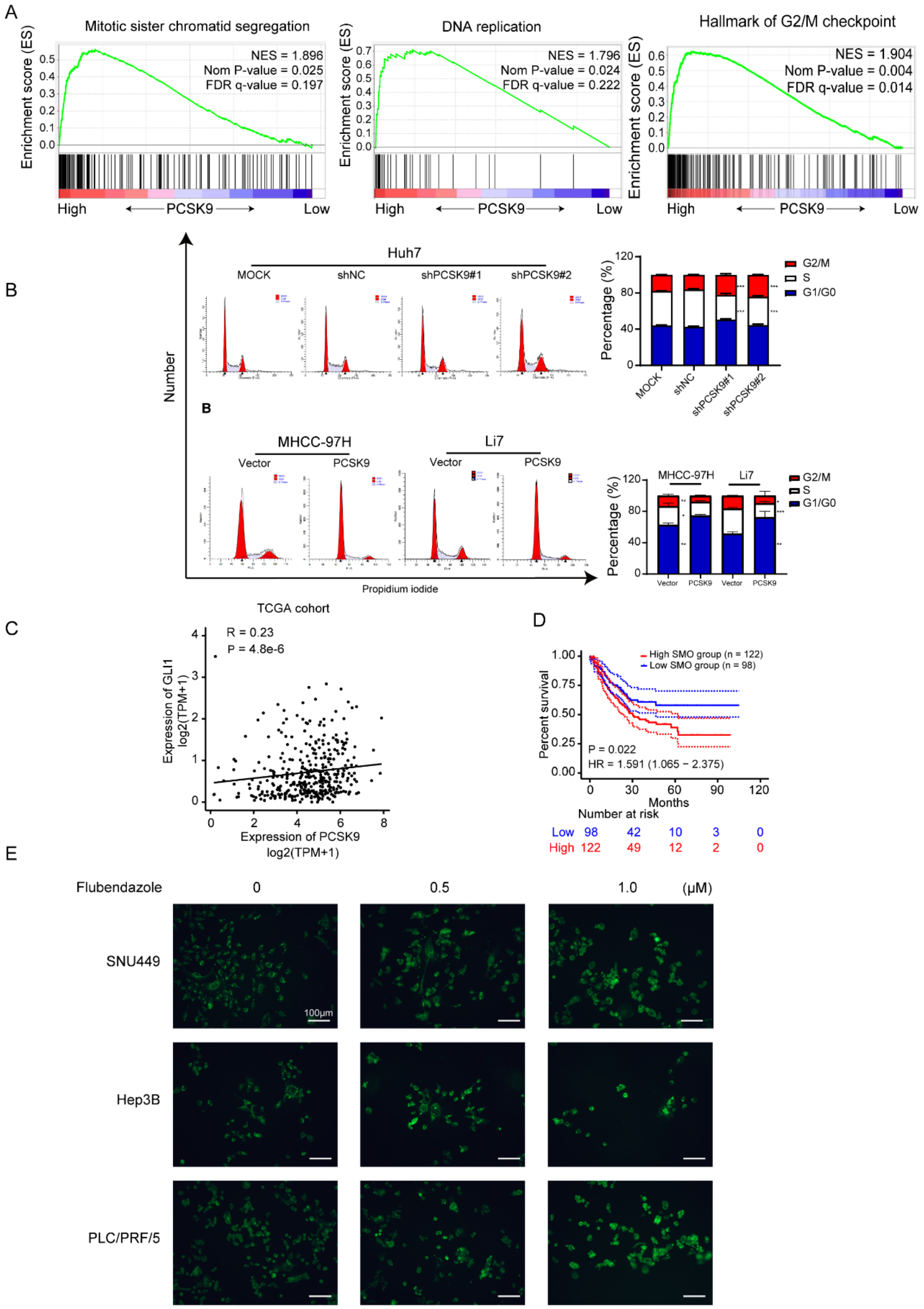

Figure S8. Flubendazole down-regulates the Hh signaling pathway by targeting PCSK9.

A GSEA analysis comparing transcriptomes of HCC cells with high or low expression of PCSK9 in TCGA database. NSE, normalized enrichment score. **B** Overexpression and knockdown of PCSK9 in HCC cells stained with PI. DNA content was quantified and shown in representative images conducted by flow cytometry analysis. **C** Correlation of mRNA expression of Gli1 and PCSK9 in TCGA database (Spearman correlation coefficient R = 0.23, $P < 0.001$). **D** Kaplan–Meier survival analysis of overall survival of HCC patients with high or low expression of SMO ($P = 0.022$, HR = 1.591) from TCGA database. **E** Fluorescence images of HCC cells treated with control (DMSO), 0.5 μM and 1 μM Flubendazole and then incubated with NBD cholesterol for 1 hour. Scale bars, 100 μm.

A

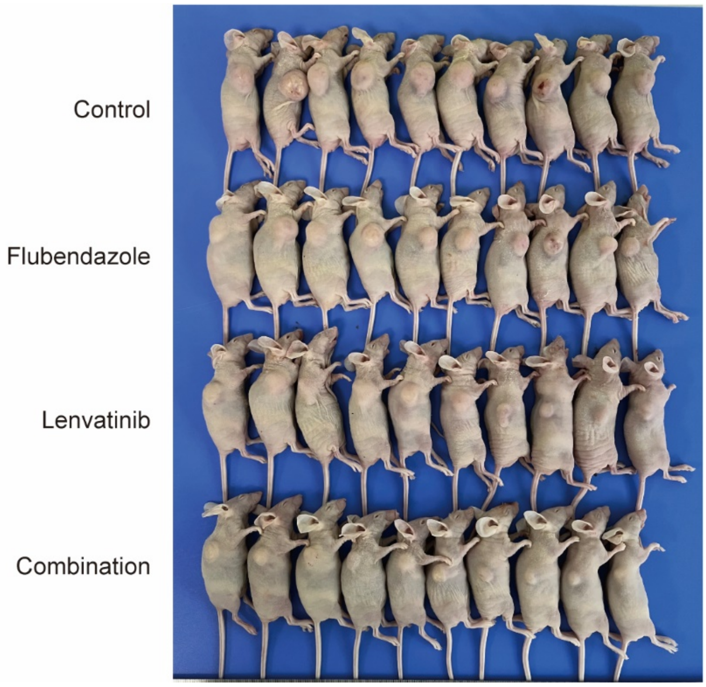

Figure. S9. Flubendazole combined with lenvatinib showed synergic effect in HCC treatment.

A The images of tumor-bearing nude mice treated with control or flubendazole alone or lenvatinib alone or combination of the two drugs.

Supplementary Tables and legends

Table S1. Target sequences of shRNA used in the study.

Target gene name	Target Sequences
**shPCSK9#1**	GCCGTAGACAACACGTGTGTA
**shPCSK9#2**	GCCAGCAAGTGTGACAGTCAT

Table S2. Primer sequences of genes used in the study.

Primers	Primer Sequences
**PCSK9-F**	CACGGAACCACAGCCACCTTC
**PCSK9-R**	TCTCCTCCTTCAGCACCACCAC
**GAPDH-F**	AGAAGGCTGGGGCTCATTTG
**GAPDH-R**	AGGGGCCATCCACAGTCTTC

Table S3. Antibodies used in western blotting and immunohistochemistry.

Antibody name for WB	Catalog	Species origin	Dilution	Company
**PCSK9**	#85813	Rabbit	1:1000	CST
**HRP-anti-Rabbit IgG**	#7074	Goat	1:5000	CST
**HRP-Anti-beta Actin**	ab8226	Mouse	1:25000	Abcam

Antibody name for IHC	Catalog	Species origin	Dilution	Company
**PCSK9（2F1）**	MA5-32843	Mouse	1:250	Invitrogen
**REAL EnVision**	Kit K5007	/	/	DAKO

Table S5. Demographic information of HCC patients in ELISA assays

**Demographic information**		
**Age**	52.18-year-old (mean)	
**Sex**	Male: 72/100 (72%)	
	Female: 28/100 (28%)	
**AFP**	711.94 (mean)	
**Tumor stage**	I	3/100 (3%)
	II	42/100 (42%)
	III	25/100 (25%)
	IV	30/100 (30%)
**Metastasis**	Intrahepatic metastasis	31/100 (28%)
	Vein	4/100 (4%)
	Vascular	6/100 (6%)
**Cirrhosis**	92/100 (92%)	
**HBV**	77/100 (77%)	

AFP: alpha fetoprotein; HBV: Hepatitis

Table S6. Coefficient of drug interaction (CDI) of flubendazole combined with lenvatinib

Flu (μM)	Len (μM)	Li7	LYHCC-10	SNU449	PLC/PRF/5	MHCC-97H	Hep3B
0.25	2.00	0.65	0.79	0.99	0.91	0.88	0.99
0.25	5.00	0.65	0.78	0.88	0.87	0.84	0.99
0.25	10.00	0.65	0.75	0.78	0.92	0.89	1.02
0.50	2.00	0.63	0.71	1.01	0.84	0.94	0.98

0.50	5.00	0.55	0.68	0.98	0.82	0.85	0.95
0.50	10.00	0.48	0.68	0.85	0.93	1.02	1.00
